# Mathematical estimation of half‐value layer thicknesses

**DOI:** 10.1002/acm2.13385

**Published:** 2021-09-01

**Authors:** Farid H. Omoumi, Xizeng Wu, Muhammad U. Ghani, Molly D. Wong, Yuhua Li, Hong Liu

**Affiliations:** ^1^ The School of Electrical and Computer Engineering The University of Oklahoma Norman Oklahoma USA; ^2^ Department of Radiology University of Alabama at Birmingham Birmingham Alabama USA

**Keywords:** half‐value layer, quarter‐value layer, tenth‐value layer, x‐ray spectrum

## Abstract

**Objective:**

The objective of this article is to introduce a simplified and swift method to satisfactorily estimate the half‐value layers (HVL), quarter‐value layer (QVL), and tenth‐value layer (TVL) from the x‐ray spectra emitted by any diagnostic radiology or kV radiotherapy x‐ray tubes.

**Methods:**

A CdTe x‐ray and Gamma detector (X‐123 CdTe, AmpTek Inc.) is used to measure the x‐ray spectra at four different x‐ray energies (low, mid, high energy x‐rays) with different external filtering. The software “*SpekCalc GUI”* (Developed in McGill University, Montreal, Canada) is also used to obtain the simulated x‐ray spectra. Both measured and simulated spectra are used to compute the HVL thicknesses of Aluminum by a mathematical method presented in this article. Next, the HVL thicknesses for corresponding tube potentials are also measured by calibrated ionization chamber and varying thicknesses of aluminum plates. Finally, the computed and measured HVL, QVL, and TVL thicknesses are compared to evaluate the efficacy of the presented method.

**Results:**

The results show acceptable concordance between computed and measured quantities. The disagreement rates between measured HVL and the values derived mathematically from the x‐ray spectra are 10 to 90 micrometers of Aluminum at tube potentials of 31 kV to 120 kV. As it is shown, a negligible discrepancy is observed between the analytical estimation and the experimental assessments.

**Conclusion:**

The HVL is an essential component in the evaluation of the quality of an x‐ray beam. However, its measurement could occasionally be challenging, time‐consuming, or uncertain due to some technical difficulties. Although the scope of this study is not to undermine the value of conventional and widely accepted practice to determine the HVL thickness, the introduced method provides the fast, more convenient, and comparably reliable technique to estimate the HVL, QVL, and TVL by employing the given x‐ray spectrum.

## 1 INTRODUCTION

The standard quality assurance and patient dose reduction closely depend on the x‐ray beam quality. Determination of the half‐value layer (HVL) is usually performed to assess the x‐ray beam quality. However, HVL or other measures like QVL and TVL are not a quick procedure, and in a few circumstances, such as computed tomography (CT) or in some Automated Exposure Control (AEC) devices, could be an arduous task. The HVL measurement in CT is usually done in nonrotating exposure mode that needs the assistance of the service engineer to disable the rotating nature of the CT device. In many newer fluoroscopy units, placing the aluminum foil in front of the source triggers the automatic tube potential and current readjustment to maintain brightness control on the detector.

Additionally, the HVL measurement must be performed under special conditions such as narrow x‐ray beam and minimal scattering from surrounding objects. Controlling the x‐ray scatter might be challenging in various equipment due to fixed objects around the device at the operational site. Moreover, it is noted by the manufacturers that the accuracy of the ionization chamber (IC) is linked to the x‐ray energies and exposure rate. The ionization chambers at the best operating condition usually carry ±4% tolerance in calibration accuracy with pre‐identified x‐ray beam hardness, as well as ±5% tolerance in x‐ray energy and exposure rate dependence. However, the increasing thicknesses of high attenuating materials such as Aluminum or Copper block the low‐energy photons and consequently alters the accuracy of the calibrated ionization chambers. Additionally, the unknown amount of x‐ray scattering passing through the varying thickness of aluminum plates, the position of the x‐ray source in the unit, and the difficulty of keeping the exposure settings constant in some equipment collectively result in technical challenges in measuring the accurate HVL, QVL, or tenth‐value layer (TVL). Bremsstrahlung x‐rays are produced by electrons striking an anode target in the x‐ray tube. In the 1920s, Hendrik Kramers discovered a formula for the continuous spectral distribution. Since the days of Kramers, many scientists have attempted to simulate the x‐ray spectra to avoid the complexity of experimental x‐ray spectroscopy.^1^, ^2^, ^3^, ^4^, ^5^, ^6^, ^7^ By advancing the simulation precision over many years, more accurate simulated x‐ray spectra are currently available and could be used with high certainty. Additionally, an authentic x‐ray spectrum can always be measured by high‐performance x‐ray spectroscopy devices.

Considering all complexities for measuring the HVL in different circumstances, we will introduce a simple analytical technique to estimate the HVL, QVL, or TVL by using the pre‐known x‐ray spectra. Presumably, this approach has been previously adopted by many scholars to estimate the HVL without measuring the real quantity through the experimental attempt. However, to the best of our knowledge, it is never explicitly elucidated in the literature. The comprehensive explanation of the procedure in this article would enable everyone to straightforwardly perform the HVL estimation, without lengthy experimental procedure but with decent precision.

## 2 MATERIALS AND METHODS

The x‐ray photons either pass through or are absorbed/scattered by the objects. The x‐ray interaction with the matter is related to the number of atoms per volume unit of the object and, consequently, the probability of photons being absorbed or scattered by that object which can be defined as the linear attenuation coefficient of the object.^8^, ^9^ The final x‐ray exposure after passing through the object can be described by the Beer‐Lambert equation as follows:

(1) $${I=I}_{0}e^{‐\mu x}$$

where, $I_{0}$ is the x‐ray exposure without interacting by attenuating object at a defined distance, I is the measured x‐ray exposure at the same plane when the object of thickness "x" is in the path of the x‐ray beam and μ is the linear attenuation coefficient of the attenuating object. Since the linear attenuation of an object heavily depends on the x‐ray photon energy, in a polychromatic x‐ray beam, the Beer‐Lambert equation can be extended as follows:

(2) $$\int IdE=\;\int I_{0(E)}e^{‐\mu_{E}\;.\;\;x}\;dE$$

where, $\mu_{E}$ is the linear attenuation coefficient of the object at the photon energy of E. Although the x‐ray absorption by air is insignificant, the photon absorption by air should also be considered for precise HVL estimation because the experimental HVL measurement is not being done in the vacuum. The x‐ray mass energy‐absorption coefficient of the air is solely used to determine the x‐ray exposure at a distance without an object in the path of the beam. The following equation can be utilized to estimate the x‐ray exposure without the attenuating object:

(3) $$I_{1}=\int E.S\left(E\right).{\left(\frac{\mu }{\rho }\right)}_{E(en‐Air)}dE$$

where, $S\left(E\right)$ is the normalized x‐ray spectrum and ${\left(\frac{\mu }{\rho }\right)}_{E(en‐Air)}$ is the mass‐energy absorption coefficient of the air for the x‐ray photon energy of E. When an x‐ray photon passes through the object (usually Aluminum to evaluate the beam quality within the diagnostic x‐ray energy), it is scattered and absorbed by the object. Therefore, the x‐ray exposure after passing through the object with thickness "x" can be determined by:

(4) $$I_{2}=\int E.S\left(E\right).{\left(\frac{\mu }{\rho }\right)}_{E(en‐Air)}.e^{‐\mu_{E}\;.\;\;x}\;dE$$

HVL is the thickness of the object that reduces the x‐ray exposure by one‐half after x‐ray photons pass through the object. By the definition of HVL, the measured value for $I_{2}$ equals one‐half of $I_{1}$. Therefore, from equations 3 and 4, the HVL can be estimated by solving equation 5 for x:

(5) $$\int E.S\left(E\right).{\left(\frac{\mu }{\rho }\right)}_{E(en‐Air)}.e^{‐\mu_{E(AL)}\;.\;\;x}\;\;dE=\frac{1}{2}\int E.S\left(E\right).{\left(\frac{\mu }{\rho }\right)}_{E(en‐Air)}dE$$

The QVL and TVL are also computable by the equation above if we solve the equation with a constant of $\frac{1}{4}$or $\frac{1}{10}$on the right side of the equation, respectively. Due to the nature of measured x‐ray spectra which they are discrete quantity rather than continuous dataset, equation five can be rewritten as a discrete equation by any desired keV interval:

(6) $$\sum E.S\left(E\right).{\left(\frac{\mu }{\rho }\right)}_{E(en‐Air)}.e^{‐\mu_{E}\;.\;\;x}=\frac{1}{2}\sum E.S\left(E\right).{\left(\frac{\mu }{\rho }\right)}_{E(en‐Air)}$$

As it is anticipated, the smaller the keV increment, the less error and uncertainty will be introduced by computation. For simplicity, we estimate the HVL, QVL, or TVL by using the normalized spectrum in energy level (E) of 1 keV intervals. The introduced error by one keV x‐ray energy gap is expected to be trivial. The linear attenuation coefficients of aluminum ($\mu_{E}$) and air mass energy‐absorption coefficients ${\left(\frac{\mu }{\rho }\right)}_{E(en‐Air)}$ for each energy level should be available for HVL calculation by this method.

The XCOM program provided by the National Institute of Standard and Technology (NIST) is utilized to extract the aluminum mass attenuation coefficients at desired x‐ray energy levels.^10^ The air mass energy‐absorption coefficients for x‐ray energies of 1keV to 20MeV are also provided by NIST.^11^ The NIST data set for air mass energy absorption coefficients does not cover all energy levels, and within the diagnostic x‐ray energy range, there are only 11 data points that are presented by the NIST. For interpolation accuracy, the air mass energy‐absorption coefficients for interested x‐ray photon energies were individually fit utilizing the least‐square technique^12^, ^13^ and the expressions:

(7) $${\left(\frac{\mu }{\rho }\right)}_{E\left(en‐Air\right)}=a_{1}+a_{2}E^{‐4.5}+a_{3}E^{‐3.5}+a_{4}E^{‐2.7}+a_{5}E^{‐1.6}$$

Where, $a_{i}$ are the constants derived from solving five equations at five different x‐ray photon energies. The calculated air mass energy absorption coefficients at various x‐ray energies and the exploited linear attenuation coefficients for the Aluminum within the diagnostic x‐ray energy range are tabulated in Appendix A.

At the initial step, we measured the x‐ray spectra at various tube potentials and filtration with the cadmium telluride (*CdTe*) x‐ray detector (X123‐CdTe Complete X‐ray & Gamma Ray detector, Amptek). The device has a detector area of 25 mm^2^, 25 µm thick graphite plus 100 µm thick Beryllium window, and it provides the channel resolution of up to 8K. The multiple layer collimator (Amptek, Bedford, MA) with stainless steel housing, Brass spacer, and two Tungsten Collimator disks are used on the spectrometer during the spectroscopy. The Tungsten disks are made of alloy HD17 (90% W, 6%Ni, and 4% Cu) with 2 mm thicknesses, 1000 µm, and 200 µm holes, respectively. The utilized spectrometer measures the received number of x‐ray photons in energy resolution of 0.04 kV. We utilized a micro‐focus X‐ray source (Hamamatsu Photonics, Model L9181‐06), which can provide up to 300 μA tube current and 130 kV tube voltage. The x‐ray source has a tungsten (W) anode target, a Beryllium output window with thickness of 0.5 mm, and provides a focal spot size of 50 μm or smaller, depending on the tube output power. The x‐ray spectrum at any tube potential is measured for five consecutive times with a resolution of 4096 channels, and the average values are reported as the ultimate result. The relevant channels to x‐ray energy interval are combined to include the total number of photons in 1 keV increment.

Two physical effects may alter the recorded raw spectrum: (a) escape of secondary x‐ray photon from the *Cd* or *Te* atoms and (b) loss of efficiency due to attenuation in the Beryllium window and the transmission through the detector. The secondary x‐ray photons, which are developed in the detector by interaction with *Cd* or *Te*, escape the detector and reduce the measured energy. The difference between the actual number and the detected number of photons, *N_∆_(E)*, at the energy of *E*, can be determined by:

(8) $$N_{\Delta }\left(E\right)=\;\sum_{p}f_{p}\left(E+E_{p}\right)N_{r}\left(E+E_{p}\right)‐\sum_{p}f_{p}\left(E‐E_{p}\right).N_{r}\left(E\right)$$

where *p* represents the four escape lines with energies $E_{p}$, $f_{p}\left(E\right)$ is the probability of characteristic photon escaping at energy *E*, and $N_{r}\left(E\right)$ is the real number of photons at the energy level of *E*. The escape peak correction is performed by stripping procedures which is discussed in detail in the literature^14^ on a channel‐by‐channel basis.

The correction tool (XRS‐FP, Amptek) utilizes the described algorithm to make the required correction on escape peak as well as efficiency loss for the recorded raw spectrum. We used the XRS‐FP software to render the real spectra from the raw spectra recorded by the spectrometer. Thereafter, measured spectra at specified tube potential or filtration are normalized by desired keV intervals. The attenuation and mass energy‐absorption coefficients are energy‐dependent, and therefore median point for each x‐ray energy level are used to roughly cancel out the variations in any given one keV range.

HVL, QVL, and TVL for any given x‐ray exposure were measured in the lab using narrow beam geometry, calibrated ionization chambers, and varying thickness of aluminum slabs.^15^, ^16^ The Dedicated Mammography Chamber (10X6‐6M, Radcal®) is used for low energy beams. The Leakage and Low‐Level Measurements Chamber (10X6‐180, Radcal®) is used for the mid‐energy and the high‐energy x‐ray beams. Incident x‐ray exposure without placing the Aluminum foil in the x‐ray beam at the distance of 69 cm is measured several times, and the average value is recorded as $I_{0}$. The varying thickness of aluminum foils are added in the x‐ray path one after another, and the average values for the x‐ray exposures are recorded for each step. The HVL, QVL, and TVL are estimated from collected x‐ray exposure data at the same plane by curve fitting.

The simulated x‐ray spectra are generated by using the software SpekCalc Pro version. This software provides the simulated x‐ray spectra emitted from a Tungsten anode. It operates between 0 and 300 kV and offers filtration of W, Cu, Al, Sn, Ta, Be, air, and water. The theoretical approach that has been used to calculate the emitted x‐ray spectra in this software was developed at the Institute of Cancer Research in UK.^17^, ^18^

**FIGURE 1:**
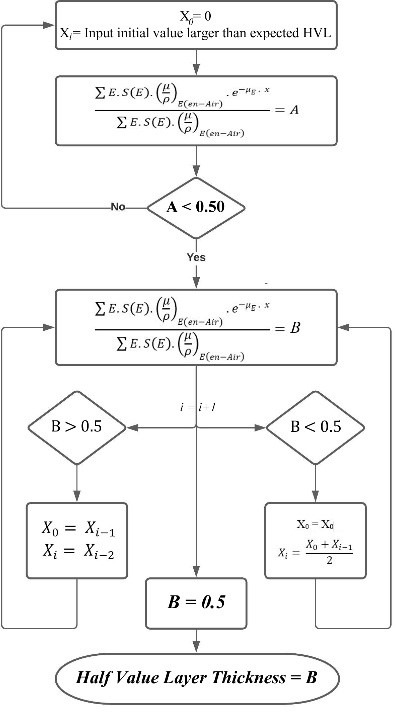
The applied numerical algorithm to solve the equation 6 for HVL thicknesses. The Value of 0.50 is replaced by 0.25 or 0.10 to estimate the QVL and TVL thicknesses, respectively

For the mathematical estimation of HVL, QVL, and TVL, solving equation 6 for "x" could be difficult and almost impractical by the analytical method. A proprietary multi‐paradigm programming language and numerical computing environment (MATLAB, MathWorks) is used to solve the equation by a numerical method, using the midpoint algorithm by iteration task. The flowchart presented in Figure 1 is the applied algorithm to solve the equation, and the source code in MATLAB is available upon request.

## 3 RESULTS

**FIGURE 2:**
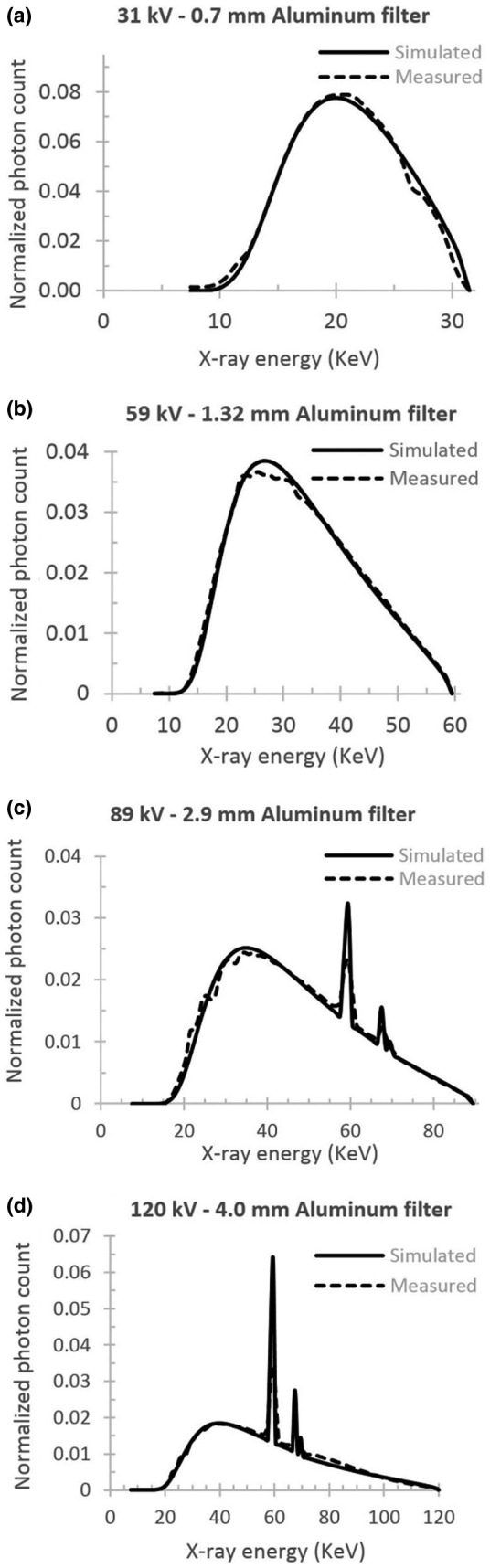
The x‐ray spectra at four different tube potentials (a:31 kV, b:59 kV, c:89 kV, and d:120 kV) measured by X‐123 CdTe, AmpTek spectrometer (Dashed line) and simulated spectra for corresponding potentials by the software SpekCalc GUI (Solid line)

**FIGURE 3:**
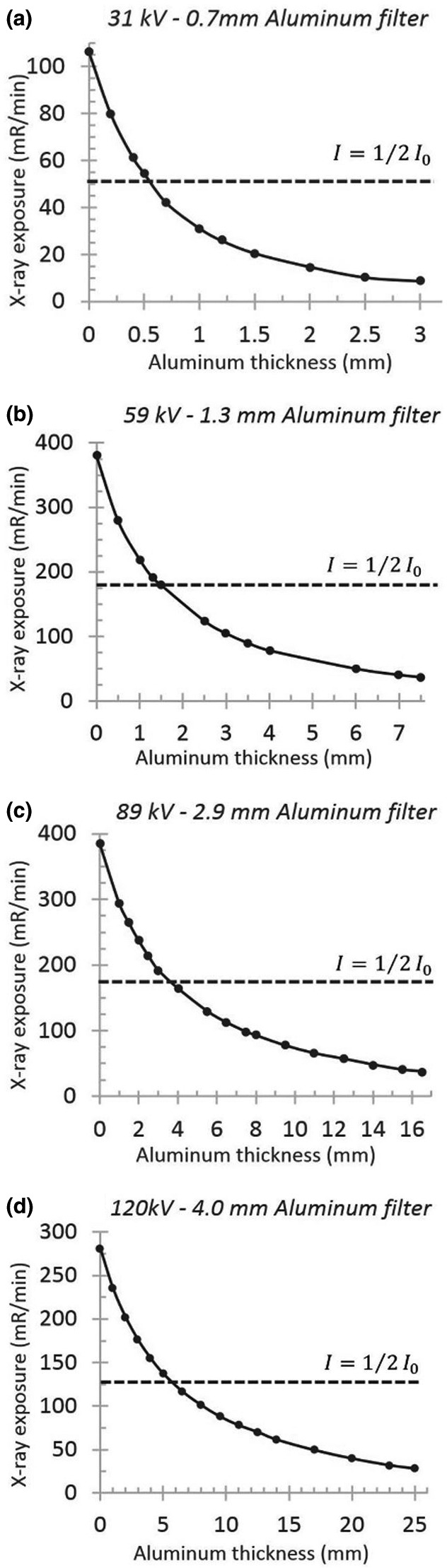
Plotted measured x‐ray exposures at four different tube potentials (a:31 kV, b:59 kV, c:89 kV, and d:120 kV) passing through varying thicknesses of aluminum foils at Source to chamber distance of 69 cm, tube currents equal to 300 µA

Figure 2 shows four normalized x‐ray spectra that were measured by Amptek x‐ray spectrometer and the corresponding spectrum simulated by SpekCalc software. The x‐ray exposures under specified conditions at the distance of 69 cm from the source are measured consequently with varying thickness of aluminum slabs. Plotted exposure values can be found in Figure 3. The horizontal dotted lines are placed at the corresponding HVL values.

The calculated HVL, QVL, and TVL derived from measured x‐ray spectra and the corresponding values measured by a calibrated ionization chamber in the experiment are presented in Table 1. The disagreement between the measured HVL and computed HVL from the measured x‐ray spectra for 31 kV and 59 kV x‐ray beams is 0.01 mm of aluminum while the disagreement between these two values for 89 kV and 120 kV x‐ray beams are 0.05 mm and 0.09 mm of aluminum, respectively.

**TABLE 1:** The measured HVL, QVL and TVL (in millimeter of aluminum) vs. corresponding estimated values by mathematical calculation, using the measured x‐ray spectra and the simulated x‐ray spectra. The examined x‐ray energies cover low, mid, and high‐energy x‐ray beams with varying external filtrations. The percentage differences between measured and calculated values are shown inside the parentheses

		31 kV *0.7 mm Al filter*	59 kV *1.3 mm Al filter*	89 kV *2.9 mm Al filter*	120 kV *4.0 mm Al filter*
Measured Values	HVL	0.51	1.31	2.99	5.05
	QVL	1.23	3.38	7.66	12.21
	TVL	2.47	7.36	16.21	25.23
Calculated by Measured Spectra	HVL	0.50 (−1.96%)	1.30 (−0.76%)	2.94 (−1.67%)	4.96 (−1.78%)
	QVL	1.22 (−0.81%)	3.33 (−1.48%)	7.57 (−1.17%)	12.35 (+1.15%)
	TVL	2.43 (−1.62%)	7.28 (−1.09%)	16.10 (−0.68%)	24.80 (−1.70%)
Calculated by Simulated Spectra	HVL	0.50 (−1.96%)	1.30 (−0.76%)	2.93 (−2.0%)	4.97 (−1.58%)
	QVL	1.22 (−0.81%)	3.33 (−1.48%)	7.57 (−1.17%)	12.30 (+0.73%)
	TVL	2.42 (−2.02%)	7.28 (−1.09%)	16.10 (−0.68%)	24.65 (−2.30%)

Table 1 also shows the computed HVL, QVL, and TVL derived from simulated x‐ray spectra in contrast to measured values by a calibrated ionization chamber for the same experimental settings. Similarly, the disagreement between the measured HVL and computed HVL from the simulated x‐ray spectra for 31 kV and 59 kV x‐ray beams is 0.01 mm of aluminum. The disagreements between these two values for 89 kV and 120 kV x‐ray beams are 0.06 mm and 0.08 mm of Aluminum, respectively. The measured and estimated values for the QVLs and TVLs for corresponding x‐ray energies are also reported in Table 1.

## 4 DISCUSSION ON CONCLUSION

The x‐ray beam quality or penetration ability of utilized radiation is usually characterized by illustrating the thickness of Aluminum or copper that reduces the intensity of x‐ray to one‐half. A polychromatic x‐ray beam is generally used in diagnostic or therapeutic radiology, and x‐ray photons at various energies are absorbed or scattered differently. Hence, the measurement of HVL under certain experimental conditions is widely accepted by medical physicists. However, the narrow beam implementation, the position and the distance of ionization chamber from the source and the attenuating material, the presence of scattering material in the vicinity of the chamber, the calibration accuracy of utilized detector, the energy dependence or the exposure rate dependence of the ionization chambers, the ability of equipment to emit stationary and constant x‐ray exposure during the experiment, etc. sometimes introduce technical challenges to readily measuring the HVLs.

On the other hand, the HVL measurement might be questionable due to the issues related to the energy dependence and the calibration accuracy of ionization chambers, while the x‐ray spectra continually become harder by adding additional aluminum foils in the HVL measuring procedure. As it is shown in the results, the calculated values for HVL, QVL, and TVLs from the x‐ray spectra are usually smaller than the measured ones. This might be expected due to various reasons, including but not limited to unknown scattering rate during the experiment or issue with calibration in the ion chamber when the beam continually gets harder during the experiment by adding the additional aluminum filters. Another concern in experimental measurement is the robustness of the curve fitting model to estimate the exact values. Theoretically, the reduction rate for the x‐ray exposure shall follow the Beer‐Lambert equation, but the calibration issue for various x‐ray spectra and the scattered x‐ray photons slightly distort the exponential curve. Hence, the applied fitting model for the generated curve may also have some impact on the HVL, QVL, and TVL estimation. For instance, in Table 1, all the measured values are greater than calculated values, with one exception of QVL for 120 kV beam. The reason could be the fitting model for the imperfect exponential curve caused by relatively thicker filtration for 120 kV beam during the experiment.

The proposed method in this article provides a consistent approach to calculate the HVL, QVL, and TVL in a variety of situations such as high‐energy x‐ray breast imaging, CT scan studies, clinical radiographic equipment with fixed AEC feature, inevitable x‐ray scattering from the surrounding objects in diagnostic radiology or radiation therapy sites, etc. with adequate precision. If the measured x‐ray spectra are being used to calculate the HVL, the accuracy of the measured spectrum must be confirmed as the spectroscopy itself is a sensitive procedure, and a well‐experienced operator is a fundamental requirement. Correspondingly, if the simulated x‐ray spectra are used to estimate the HVL by this method, the robustness of the x‐ray simulating method shall be upheld to avoid any imprecise results.

Needless to point out, the conventional HVL measurement is widely accepted in the field, and the scope of this work is not to undermine the current practice or to suggest replacing it with the mathematical HVL estimation. Additionally, the novel solid‐state diagnostic dosimeters are increasingly being used in clinical practice. The significant advantage of these dosimeters over the traditional ionization chambers is their capability to identify the air kerma, tube voltage, exposure time, and HVL from single irradiation, and accommodate the problems associated with the backscatter radiation. However, a relatively noticeable error is expected when measurement involves the x‐ray beams filtered by various materials, Copper, for instance.^19^ Nevertheless, this straightforward method could be an appropriate solution and provides a fast and convenient estimation of HVL, QVL, or TVL with decent precision, when the HVL measurement is challenging due to given conditions or expresses the possibility of disputable values if the measured or simulated x‐ray spectra are available.

## CONFLICT OF INTEREST

We have no conflict of interest to disclose.

## AUTHOR CONTRIBUTIONS

Farid H Omoumi: Contributed in all aspects of the research, including the data acquisition, development of the numerical solution, drafting the manuscript. Xizeng Wu: Contributed in all aspects of the research, theoretical development, and/or revised the manuscript. Xizeng Wu just retired from the Department of Radiology at the University of Alabama. Muhammad U Ghani: Contributed in data acquisition and/or revised the manuscript. Molly D Wong: Contributed in data acquisition, and/or revised the manuscript. Yuhua Li: Contributed in data acquisition, and/or revised the manuscript. Hong Liu: Contributed in all aspects of the research, theoretical development, and/or revised the manuscript.

## Appendix A Dry air mass‐energy absorption and Aluminum linear attenuation coefficients 7.5 keV to 149.5 keV

**Table:**

E (KeV)	${\left(\frac{\mu }{\rho }\right)}_{E\left(Air\right)}$(cm^2^/g)	μ_Al_ (cm^−1^)
7.5	1.151E+01	1.638E+02
8.5	7.839E+00	1.138E+02
9.5	5.559E+00	8.224E+01
10.5	4.075E+00	6.129E+01
11.5	3.069E+00	4.691E+01
12.5	2.365E+00	3.671E+01
13.5	1.858E+00	2.926E+01
14.5	1.484E+00	2.372E+01
15.5	1.203E+00	1.950E+01
16.5	9.879E−01	1.624E+01
17.5	8.206E−01	1.367E+01
18.5	6.888E−01	1.164E+01
19.5	5.836E−01	9.992E+00
20.5	4.986E−01	8.656E+00
21.5	4.294E−01	7.555E+00
22.5	3.724E−01	6.642E+00
23.5	3.251E−01	5.881E+00
24.5	2.855E−01	5.239E+00
25.5	2.522E−01	4.694E+00
26.5	2.240E−01	4.227E+00
27.5	2.000E−01	3.827E+00
28.5	1.794E−01	3.482E+00
29.5	1.616E−01	3.179E+00
30.5	1.463E−01	2.918E+00
31.5	1.330E−01	2.689E+00
32.5	1.213E−01	2.494E+00
33.5	1.111E−01	2.308E+00
34.5	1.022E−01	2.150E+00
35.5	9.425E−02	2.009E+00
36.5	8.725E−02	1.883E+00
37.5	8.104E−02	1.770E+00
38.5	7.552E−02	1.668E+00
39.5	7.059E−02	1.576E+00
40.5	6.619E−02	1.494E+00
41.5	6.224E−02	1.419E+00
42.5	5.869E−02	1.351E+00
43.5	5.549E−02	1.289E+00
44.5	5.261E−02	1.232E+00
45.5	5.001E−02	1.180E+00
46.5	4.765E−02	1.132E+00
47.5	4.551E−02	1.089E+00
48.5	4.357E−02	1.048E+00
49.5	4.180E−02	1.011E+00
50.5	4.020E−02	9.768E−01
51.5	3.873E−02	9.449E−01
52.5	3.740E−02	9.155E−01
53.5	3.618E−02	8.882E−01
54.5	3.507E−02	8.626E−01
55.5	3.404E−02	8.388E−01
56.5	3.311E−02	8.167E−01
57.5	3.225E−02	7.959E−01
58.5	3.147E−02	7.765E−01
59.5	3.075E−02	7.584E−01
60.5	3.009E−02	7.414E−01
61.5	2.948E−02	7.255E−01
62.5	2.892E−02	7.104E−01
63.5	2.841E−02	6.961E−01
64.5	2.794E−02	6.828E−01
65.5	2.750E−02	6.702E−01
66.5	2.711E−02	6.580E−01
67.5	2.674E−02	6.470E−01
68.5	2.641E−02	6.362E−01
69.5	2.610E−02	6.259E−01
70.5	2.581E−02	6.162E−01
71.5	2.555E−02	6.070E−01
72.5	2.532E−02	5.984E−01
73.5	2.510E−02	5.900E−01
74.5	2.490E−02	5.822E−01
75.5	2.471E−02	5.746E−01
76.5	2.455E−02	5.673E−01
77.5	2.439E−02	5.606E−01
78.5	2.426E−02	5.538E−01
79.5	2.413E−02	5.476E−01
80.5	2.401E−02	5.417E−01
81.5	2.391E−02	5.358E−01
82.5	2.382E−02	5.304E−01
83.5	2.373E−02	5.250E−01
84.5	2.366E−02	5.198E−01
85.5	2.359E−02	5.150E−01
86.5	2.353E−02	5.101E−01
87.5	2.348E−02	5.055E−01
88.5	2.343E−02	5.012E−01
89.5	2.339E−02	4.969E−01
90.5	2.336E−02	4.928E−01
91.5	2.333E−02	4.888E−01
92.5	2.330E−02	4.850E−01
93.5	2.329E−02	4.812E−01
94.5	2.327E−02	4.777E−01
95.5	2.326E−02	4.742E−01
96.5	2.325E−02	4.710E−01
97.5	2.325E−02	4.677E−01
98.5	2.325E−02	4.645E−01
99.5	2.325E−02	4.615E−01
100.5	2.325E−02	4.586E−01
101.5	2.326E−02	4.556E−01
102.5	2.327E−02	4.529E−01
103.5	2.328E−02	4.502E−01
104.5	2.330E−02	4.475E−01
105.5	2.331E−02	4.448E−01
106.5	2.333E−02	4.424E−01
107.5	2.335E−02	4.399E−01
108.5	2.337E−02	4.375E−01
109.5	2.340E−02	4.351E−01
110.5	2.342E−02	4.329E−01
111.5	2.344E−02	4.308E−01
112.5	2.347E−02	4.286E−01
113.5	2.350E−02	4.264E−01
114.5	2.353E−02	4.243E−01
115.5	2.356E−02	4.224E−01
116.5	2.359E−02	4.205E−01
117.5	2.362E−02	4.186E−01
118.5	2.365E−02	4.167E−01
119.5	2.368E−02	4.148E−01
120.5	2.371E−02	4.129E−01
121.5	2.375E−02	4.111E−01
122.5	2.378E−02	4.094E−01
123.5	2.382E−02	4.078E−01
124.5	2.385E−02	4.062E−01
125.5	2.389E−02	4.046E−01
126.5	2.392E−02	4.030E−01
127.5	2.396E−02	4.013E−01
128.5	2.399E−02	3.997E−01
129.5	2.403E−02	3.981E−01
130.5	2.406E−02	3.968E−01
131.5	2.410E−02	3.954E−01
132.5	2.414E−02	3.938E−01
133.5	2.417E−02	3.924E−01
134.5	2.421E−02	3.911E−01
135.5	2.425E−02	3.897E−01
136.5	2.428E−02	3.884E−01
137.5	2.432E−02	3.870E−01
138.5	2.436E−02	3.857E−01
139.5	2.439E−02	3.843E−01
140.5	2.443E−02	3.833E−01
141.5	2.447E−02	3.819E−01
142.5	2.450E−02	3.806E−01
143.5	2.454E−02	3.795E−01
144.5	2.458E−02	2.807E−01
145.5	2.461E−02	3.771E−01
146.5	2.465E−02	3.760E−01
147.5	2.469E−02	3.749E−01
148.5	2.472E−02	3.738E−01
149.5	2.476E−02	3.725E−01
